# Uncertainty Evaluation of a Gas Turbine Model Based on a Nonlinear Autoregressive Exogenous Model and Monte Carlo Dropout

**DOI:** 10.3390/s24020465

**Published:** 2024-01-12

**Authors:** Armando Cajahuaringa, Rubén Aquize Palacios, Juan M. Mauricio Villanueva, Aurelio Morales-Villanueva, José Machuca, Juan Contreras, Kiara Rodríguez Bautista

**Affiliations:** 1 Universidad Nacional de Ingeniería, Av. Tupac Amaru 210, Rimac, Lima 150101, Peru; acajahuaringa@uni.edu.pe (A.C.); raquize@uni.edu.pe (R.A.P.); amorales@uni.edu.pe (A.M.-V.); jmachuca@uni.edu.pe (J.M.); jcontreras@uni.edu.pe (J.C.); krodriguezb@uni.pe (K.R.B.); 2 Universidade Federal da Paraíba Campus I, Joao Pessoa 58051-900, PB, Brazil

**Keywords:** gas turbine model, artificial neural networks, Monte Carlo dropout, uncertainties

## Abstract

Gas turbines are thermoelectric plants with various applications, such as large-scale electricity production, petrochemical industry, and steam generation. In order to optimize the operation of a gas turbine, it is necessary to develop system identification models that allow for the development of studies and analyses to increase the system’s reliability. Current strategies for modeling complex and non-linear systems can be based on artificial intelligence techniques, using autoregressive neural networks of the NARX and LSTM type. In this context, this work aims to develop a model of a gas turbine capable of estimating the rotation speed of the turbine and simultaneously estimating the uncertainty associated with the estimation. These methodologies are based on artificial neural networks and the Monte Carlo dropout simulation method. The results were obtained from experimental data from a 215 MW gas turbine, getting the best model with a MAPE of 0.02% and an uncertainty associated with the turbine rotation speed of 2.2 RPM.

## 1. Introduction

Gas turbines (GT) are critical thermoelectric systems used in various industrial processes, including power generation systems. These thermoelectric plants have the characteristics of compact projects, simple installation processes, and a lower level of pollution. However, effective maintenance, monitoring, and diagnostic strategies for the GT must be carried out to achieve these objectives [1].

On the other hand, the demand for energy production is constantly increasing, and many countries are developing strategies for constructing GTs using new technologies. As a direct consequence, this demand has a high consumption of natural gas. In this way, various techniques have been developed in the scientific literature to optimize the operation of GTs, including improvements in fuel-burning modes, exhaust gas purification technologies, and modeling strategies based on data and artificial intelligence [2].

Furthermore, the introduction of modern strategies for the construction of GT models allows improvements in the reliability of these systems [3], such as the use of computational intelligence techniques and analysis and propagation of uncertainties based on fuzzy logic, artificial neural networks, and evolutionary algorithms.

Therefore, power generation systems based on GTs, which use updated methodologies, represent challenges and opportunities for optimizing the operation of GTs. Consequently, problems related to the non-linear behavior of GTs, such as uncertainties associated with the measurement of signals, and system failures are solved using these modern strategies [4].

In this sense, the objective of this work is to develop a GT model using artificial intelligence techniques, specifically NARX neural networks and LSTM models. This model considers as output the estimation of the rotational speed of the turbine, correlating with variables such as temperature, pressure, fuel flow, and power. In order to evaluate the uncertainties associated with the GT model, we used the Monte Carlo dropout simulation method, which allowed us to obtain confidence intervals related to the estimations of the turbine rotational speed.

There are some prior works related to evaluating the uncertainties in non-linear systems using different models such as neural networks (NNs) and combinations of non-linear autoregressive with exogenous input (NARX) with other several techniques to minimize the uncertainties of these systems.

For example, the authors in [5] proposed a numerical approach to overcome the accuracy of the polynomial chaos expansions (PCEs) that cannot represent the long-term dynamics of a system, by combining PCEs with non-linear autoregressive exogenous (NARX) models, producing PC-NARCX models for nonlinear systems with uncertainties subject to non-stationary stochastic excitations. The authors introduced the least angle regression (LAR) technique for computing the PC-NARX models and the proposed approach was validated with three case studies, where the authors also performed a comparison with Monte Carlo simulation and regular PCEs, to demonstrate the effectiveness of the authors’ approach.

Also, in [6], Malmström used neural networks (NNs) as a black-box model for non-linear system identification in order to improve the uncertainty in predictions by performing a linearization of the model. Then, the author investigated the uncertainty in predictions generated using different models as part of the experimental study. In the end, the experimental results showed that the linearization method produced similar results for predictions of the uncertainty using NNs, compared to existing methods.

On the other hand, the authors in [7] continued and extended prior work on NARX formulation based on Gaussian Process (GP) regression that derived expressions for Higher-order Frequency Response Functions (HFRFs) in order to visualize how different frequencies at the input to a nonlinear system interact in forming the output. The work in [7] extended the GP-NARX and converted GP prediction bounds in the time domain onto HFRFs, using as a case study an asymmetric Duffing oscillator where the authors used a simple Monte Carlo simulation in order to convert GP-NARX data into HFRFs, taking into account the uncertainty in the original GP fit and obtaining a very low uncertainty.

Additionally, in reference [8], a KNARX model was introduced for quantifying uncertainty in sparse-type nonlinear stochastic dynamical systems. This model effectively captures system nonlinearity using the NARX framework, integrates the Kriging surrogate model to handle parameter uncertainty propagation, and employs the Least Angle Regression Algorithm (LARS) to enhance computational efficiency. The study analyzed three instances of nonlinear stochastic dynamical systems, predicting time-dependent mean and standard deviation, instantaneous stochastic response characteristics, and maximum absolute response. Efficiency was gauged through CPU time and the number of required evaluations of the surrogate model. Uncertainty results were rigorously compared using Monte Carlo Simulation (MCS) and mean error, showcasing consistently excellent performance compared to MCS outcomes.

Furthermore, in a preceding study [9], the authors delved into the precise probabilistic prediction of the remaining useful life (RUL) of bearings—an imperative aspect for ensuring the safe operation of wind turbines and facilitating maintenance decision-making. The proposed probabilistic RUL prediction method introduced the PDAGRU prediction model, incorporating a parallel GRU structure with a dual-stage attention mechanism and a non-parametric uncertainty quantification approach. This novel method addressed inherent limitations, improving prediction accuracy and enabling effective uncertainty quantification. Leveraging kernel density estimation (KDE) and Monte Carlo (MC) dropout, the approach requires less prior knowledge, offering a practical and reliable means to enhance wind turbine operational reliability.

In another antecedent work [10], the authors addressed challenges arising from integrating distributed generators utilizing photovoltaic panels into distribution networks. Such integration can lead to overvoltages, undervoltages, frequency oscillations, and alterations in protection design. The study evaluated seven training algorithms within artificial neural networks (ANNs) employing NARX architecture to estimate active generated power. The statistical results were compared against support vector machine (SVM) and Kalman filter (KF) techniques. The analysis revealed that the optimal ANN training algorithm achieved a mean absolute percentage error (MAPE) of 0.02%, outperforming SVM (0.33%) and KF (3.41%). The conclusion was that ANNs proved more suitable for addressing this specific problem than the SVM and KF methodologies.

Moreover, in [11], the authors developed a methodology for the load forecasting, planning, and optimization of the operation of large-scale power systems, for a forecast horizon of 24 h. These models considered the influence of load, climatic (temperature, relative humidity), and temporal (month, day, and hour) measurement variables. The developed models used NARX topologies to find the best forecast model. Based on statistical tests, the authors reached a 95% confidence interval, which was used as a metric to assess model uncertainties.

Also, in [12], a wavelet neural network (WNN) was used to predict the exhaust emissions of an ignition engine. In this way, the WNN model established the relationship between power and fuel consumption for different speeds. The used model was improved using the stochastic gradient algorithm, showing that the results of this improved model presented high accuracy to estimate engine exhaust emissions.

Additionally, in [13], a methodology was developed for the price forecasting of Mexican red lobsters using artificial neural networks. In this way, the methodology had a contribution in supporting the price decisions of the fishermen of that region, since the uncertainties directly influence the definition of the prices. The used method was based on NARX, ARIMAX, and NAR autoregressive models, obtaining results with acceptable performance for the forecast models.

Although all of these prior works analyzed how to measure and minimize the uncertainties in non-linear systems using different combinations of black-box models, none considered a GT as a non-linear system and a time-variant system. Then, in this work, we present for the first time, to the best of our knowledge, the development of a GT model using artificial neural networks and, simultaneously, we use the Monte Carlo dropout method to obtain the estimation of the uncertainty of the GT rotational speed. Our evaluations made it possible to define confidence intervals for the model results, thus propagating the uncertainties of the measurements of the input signals to the output. In this way, the practical estimation of the uncertainties made it possible to quantify the degree of accuracy of the proposed model.

Besides, there are different motivations for modeling a GT, e.g., for GT condition monitoring, GT fault detection and diagnosis, sensor validation, system identification, GT control system design and optimization, etc. [14,15], so it is necessary to clearly establish the goal of modeling the GT for this model to be successful. In our case, our work is motivated to develop a model for GT system identification.

On the other hand, GT models based on Digital Twin (DG) are also developed to optimize the operation of the GT. These models use real-time data to update the DG, and from this representation it is possible to optimize the operation of the GT, such as determining the set point with the highest performance and evaluation for predictive maintenance, among others [16]. However, this work does not aim to develop a GT model for this type of monitoring.

The main contributions of this work are as follows: (a) the modeling of a GT using NARX and LSTM models to obtain the estimation of the rotational speed of the GT from experimental data and (b) the evaluation of the uncertainties related to the estimation of the rotational speed of the GT using the Monte Carlo dropout simulation.

This article is organized into four sections. Section 2 presents the NARX model applied to a GT. Section 3 presents the uncertainty evaluation procedure based on the Monte Carlo dropout simulation. In Section 4, we show the computational results of the developed model and the evaluation of the uncertainty. And finally, in Section 5, the conclusions and future work are presented.

## 2. Gas Turbine Model Based on NARX

The GT that will be modeled in this work is a Siemens brand, model SGT6-5000F of 215 MW, with a rotational speed of 3600 rpm, single axis, for heavy duty.

**Figure 1:**
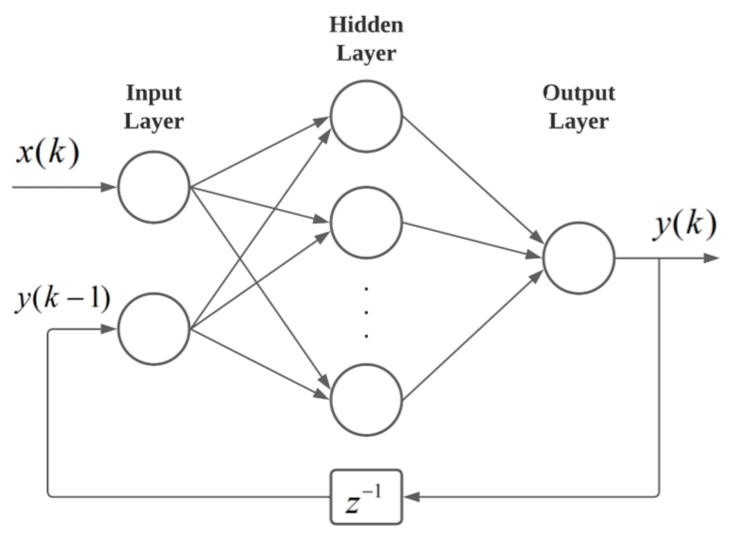
NARX neural network.

The model developed for the GT is based on a NARX-type ANN, whose architecture is illustrated in Figure 1. These neural networks are suitable for capturing the dynamics of complicated systems such as GTs [17].

The NARX are dynamic recurrent networks that correlate the current value assumed by an output parameter *y*(*t*) in a time series with the past values of the same parameter and the other inputs [18], that is, the output is a function of the *k* samples of the current and/or past inputs with the past outputs as shown in Equation (1).

*y*(*k*) = *f*(*x*(*k* − 1), …, *x*(*k* − *n_i_*), *y*(*k* − 1), …, *y*(*k* − *n_o_*)) (1)

where *n_i_* and *n_o_* are the number of samples passed in and out, respectively.

The selected GT input and output variables are shown in Table 1.

**Figure 2:**
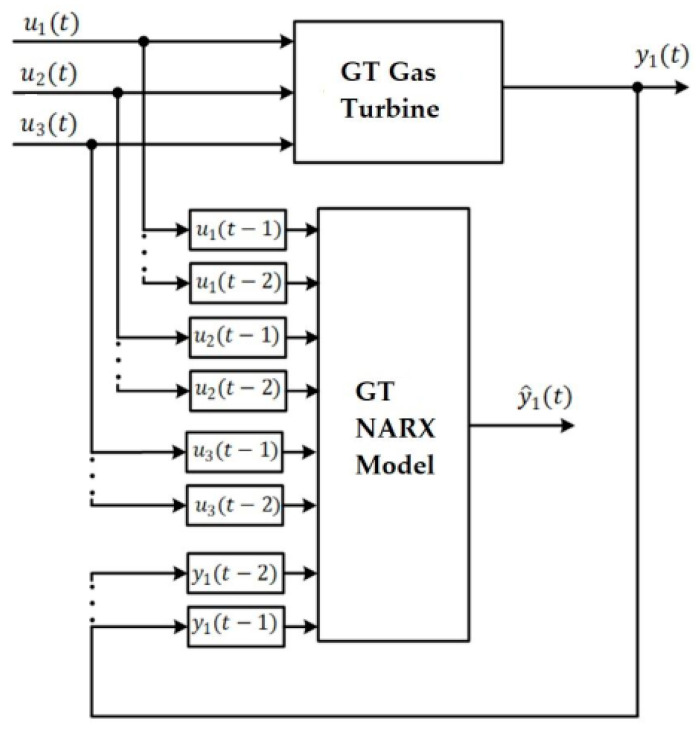
NARX model for the GT.

The NARX architecture adopted for the GT, according to Figure 1 and Equation (1), is shown in Figure 2, where $y_{1}(t)$ and ${\hat{y}}_{1}(t)$ are the current and estimated rotational speed, respectively.

The dataset used, 2305 records, was normalized in the range of [0,1] and divided into three parts: 70%, 15%, and 15% for training, validation, and testing, respectively.

In order to develop the GT NARX model, the authors used the MATLAB computational tool (V.20-22a). The variable selection procedure determined a model with three inputs and one output variable. The search for the best network took into account the variation of one or two layers, one or more lags on the input and output variables, and the configuration of hyperparameters (number of epochs of 100, learning rate of 0.01, momentum rate of 0.9, and desired error of no more than 1%).

Following exhaustive experimentation, the NARX model depicted in Figure 2, referred to as the optimal NARX, was derived. This optimal model exhibited the following hyperparameters: 2 input and output delays, 2 hidden layers, 15 neurons in the first layer, 3 neurons in the second layer, Trainlm transfer function, Logsig activation function with values between 0 and 1, 100 epochs, a desired final error of 10^−4^, a learning rate of 0.01, a momentum rate of 0.9, and a minimum relative absolute error of 1%.

The optimal NARX model, obtained through the proposed methodology, achieved performance metrics, reaching an MSE of 1.9459 × 10^−5^ by the 17th epoch, an RMSE of 0.4411%, and a MAPE of 0.0643.

## 3. Uncertainty Assessment Procedure

**Figure 3:**
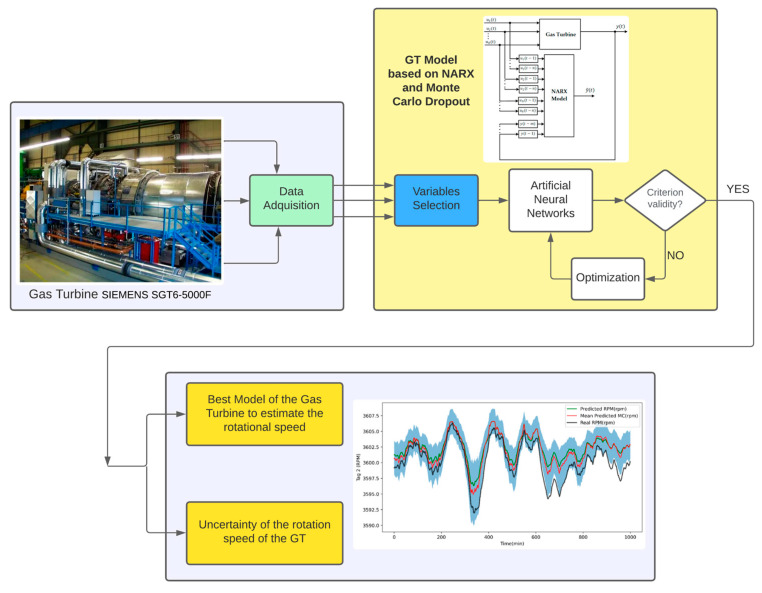
Proposed methodology for the estimation of rotational speed and analysis of uncertainty.

In this section, we present the fundamentals for the implementation of the uncertainty evaluation procedure using the Monte Carlo dropout simulation method. In Figure 3, the depicted diagram outlines the proposed methodology for estimating the rotational speed of the GT and assessing its uncertainty. The process initiates with the acquisition of data from the GT, which captures the system’s dynamic response. Following this, we use an artificial neural network to construct a model for the GT, aiming to estimate both the rotational speed and its associated uncertainty, based on a NARX-type neural network and the Monte Carlo dropout simulation method.

### 3.1. Dropout as a Regularization Technique

**Figure 4:**
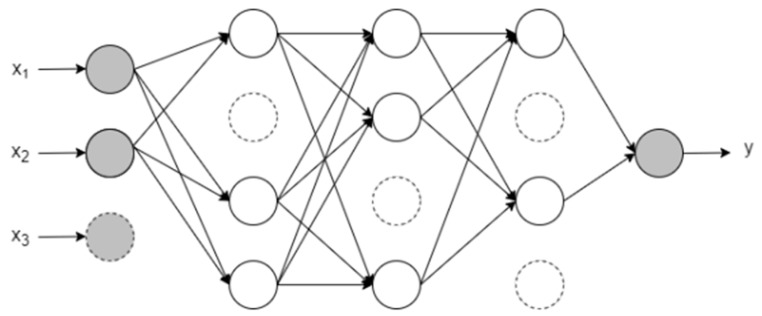
Artificial neural network after the dropout application.

According to reference [19], the dropout technique was developed in order to avoid overfitting in artificial neural networks. The method consists of temporarily removing neurons from the neural network’s layers (visible or hidden) and their input and output connections, as illustrated in Figure 4. As the dropout is a stochastic regularization technique, neurons are randomly removed from the network during the training phase, followed by a probability *p*. In the test phase, the output weights of each neuron are multiplied by *p*.

In this way, the technique provides new topologies, more optimized in the same neural network with each deactivation of neurons, since new weights will be obtained, generating new outputs. A network with *n* units (neurons) can be seen as a set of 2*^n^* optimized sub-networks [20].

### 3.2. Uncertainties in Deep Learning Models

The sources of uncertainties in a model can have various origins, such as noise in the data, lack of information about the inputs, missing data, calibration, and change in hyperparameters, among others. Such uncertainties can generate inconsistencies and loss of reliability in models based on artificial intelligence. Therefore, classifying and quantifying uncertainties in data-based models is very important, mainly when associated with tools to support decision-making and operations planning.

Two types of uncertainties in deep learning are used as a basis for most estimation cases: epistemic and random uncertainty.

Epistemic uncertainty describes the degree of confidence in the estimation results, and its main cause is the absence of model training data. Therefore, the greater the amount of input data, the lower the epistemic uncertainty. Epistemic uncertainty is the uncertainty directly linked to the model.

The random uncertainty is directly related to the input data. Therefore, data with noise and high variance are the main sources of this type of uncertainty. In this case, more than adding more data is needed to reduce the value of it.

Thus, of the types of uncertainty presented, epistemic uncertainty is the most challenging to obtain, given that it is directly linked to the model and its learning process. In order to obtain the uncertainty of the model, strategies such as training a Bayesian neural network are used, in contrast to conventional ANN, whose characteristic is purely deterministic. In reference [20], a low-computational-cost strategy was presented to obtain uncertainty in (epistemic) models based on artificial neural networks using the dropout technique, observing it from a Bayesian perspective.

### 3.3. Uncertainties Estimation

The process of obtaining uncertainties is to keep the dropout active during the testing phase and to get *T* forecast values for each sample in the artificial neural network This process of obtaining values is carried out by randomly sweeping the network *T* times and collecting the samples. In this way, the technique is known as Monte Carlo dropout. In addition, the sweeps performed in the neural network are known as forward passes. Second [20], the mean of the measurements can be written from Equation (2).

(2) $$E\left(\hat{y}\right)=\frac{1}{T}\sum_{t=1}^{T}{\hat{y}}_{t}$$

where $\hat{y}$*_t_* is each forecast sampled in the *T* forward pass by the network. Furthermore, the variance can be determined from Equation (3).

(3) $$Var\left(\hat{y}\right)\approx \tau^{-1}+\frac{1}{T}\sum_{t=1}^{T}{\hat{y}}_{t}^{T}{\hat{y}}_{t}-E{\left({\hat{y}}_{t}\right)}^{T}E\left({\hat{y}}_{t}\right)$$

where *τ* is a measure of precision, being represented by Equation (4).

(4) $$\tau =\frac{pl^{2}}{2N\lambda }$$

Thus, for each point, the estimate for the mean and variance can be determined, which will be the measure of uncertainties. The literature adopts variance as the main measure of uncertainties. However, the standard deviation can also be assumed as uncertainty when calculating the square root of the variance obtained after *T* forward passes.

## 4. Results

In this section, results of the estimation of the rotation speed of the GT will be presented considering two models developed in this work, using artificial neural networks, being the architectures used NARX and LSTM. Using the proposed methodology and from the selection of the best topology, uncertainty analyses of the model based on the Monte Carlo dropout simulation will be performed.

### 4.1. Comparison between NARX and LSTM for Rotation Speed Estimation

**Figure 5:**
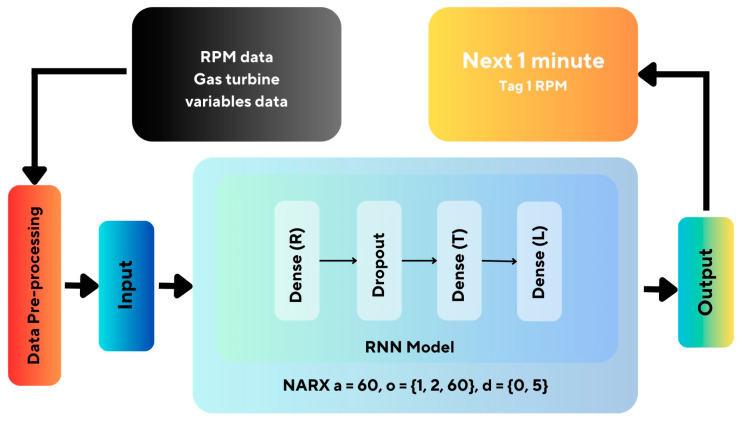
An overview of neural network recurrent NARX proposed layers.

In this section, a comparison of the two models developed for the estimation of the RPM of the gas turbine will be made, being the NARX and LSTM topologies. Figure 5 illustrates the layered model of the proposed methodology, considering the recurrent neural network with NARX architecture, where Dense (R) is the dense layer with a ReLu activation function, Dense (T) is the dense layer with a Tanh activation function, and Dense (L) is the dense layer with a Linear activation function. This topology includes the dropout analysis methodology as an uncertainty assessment tool.

For the development of the NARX network, data pre-processing methods were initially used. This stage included the imputation of missing data and data normalization. The consolidated data bank was divided into training data (70%) and test data (30%). The NARX model improves input using a recurrent neural network with self-order, exogenous order, and delay. The neural network adjusts the features to develop a time series predictor, capturing the short-term temporal relationship between the input variables and the target in more detail.

Considering the model presented in Figure 5, three models were used for simulation: NARX-RNN (recurrent neural network), NARX-LSTM, and LSTM. These models will estimate the RPM of the gas turbine, whose hyperparameter configuration is contained in Table 2.

The metrics used for the analysis and evaluation of the model were the mean square error (MSE), mean average error (MAE), and mean average precision error (MAPE). Time series cross-validation has been implemented, so the average of the metrics calculated in each *k*-fold is presented in Table 3.

**Figure 6:**
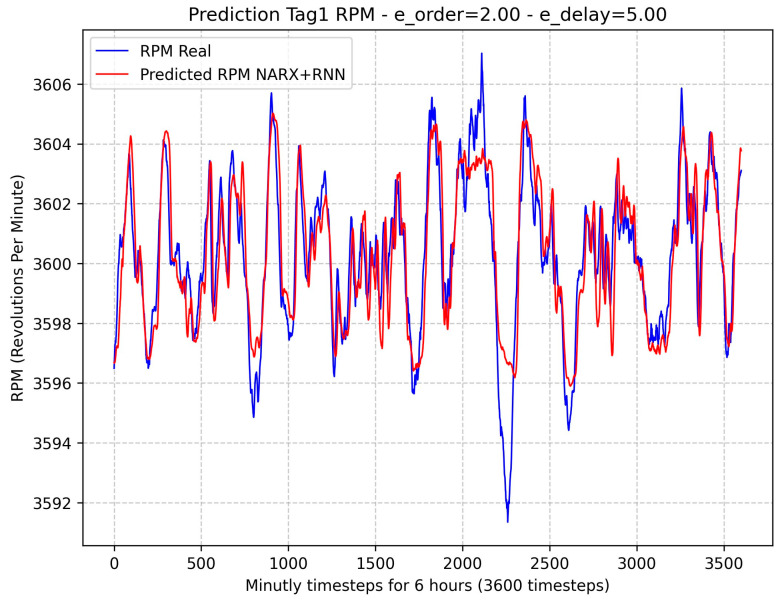
Comparison between predicted and actual values of the rotation speed (RPM) using NARX model.

Figure 6 shows the result of the NARX + RNN recurrent neural network model to estimate the rotation speed of the turbine (Tag1 RPM), with a value of MAPE equal to 0.02%. Although the neural network was trained with a history of GT data, when tested with entirely new data, it may result in a prediction with a high error, as is the case with timesteps between 2000 and 2500. This behavior is typical of highly non-linear systems, as with GT.

**Figure 7:**
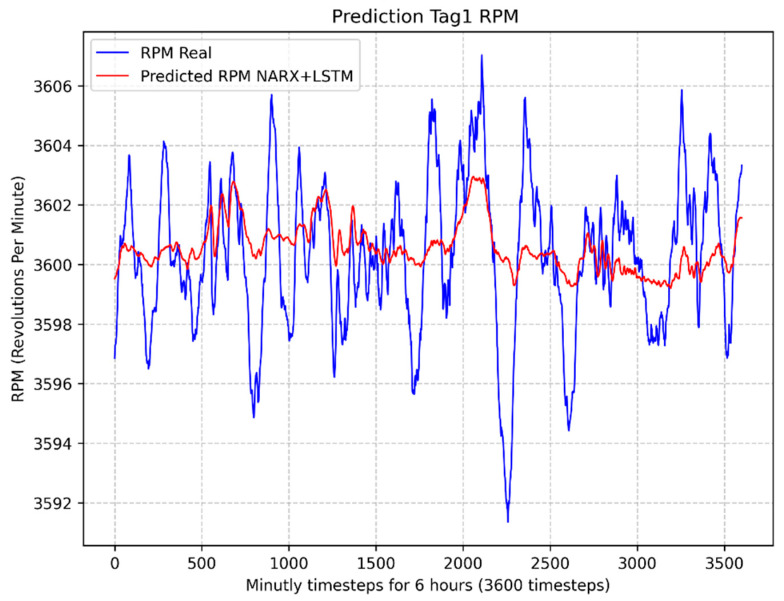
Comparison between predicted and actual values of the rotation speed (RPM) using the NARX + LSTM model.

**Figure 8:**
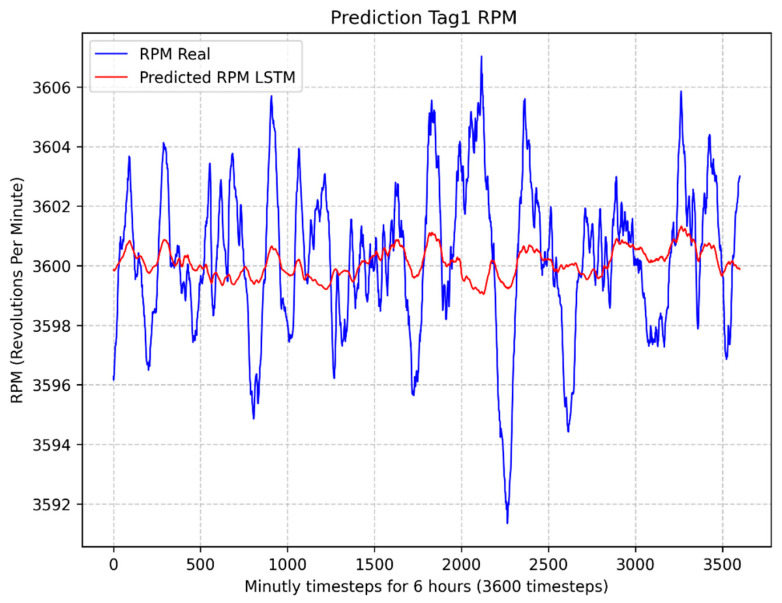
Comparison between predicted and actual values of the rotation speed (RPM) using the LSTM model.

In Figure 7 illustrates the result of the NARX + LSTM model to estimate the turbine rotation speed (Tag1 RPM), resulting in a MAPE value of 0.04%. In Figure 8 illustrates the result of the LSTM model to estimate the turbine rotation speed (Tag1 RPM), resulting in a MAPE value of 0.05%.

The results obtained in this section conclude that the winning model is the NARX, with a MAPE equal to 0.02%, lower than the other models developed (NARX + LSTM and LSTM).

### 4.2. Uncertainty Evaluation of the Rotational Speed Estimation Using the NARX Model

This study employed the NARX (nonlinear autoregressive with exogenous inputsthe) model, a neural network architecture widely used for time series forecasting. We incorporated Monte Carlo dropout during the evaluation phase to account for uncertainty in the model predictions. First, the NARX model was trained on historical time series data and exogenous inputs to predict the next value in the time series. During inference, dropout was applied, randomly dropping out neurons to estimate uncertainty. Multiple forward passes were performed using dropout, resulting in a collection of predictions. We calculated uncertainty measures such as variance or standard deviation across the predictions by employing Monte Carlo sampling. These measures provided insights into the model’s confidence in its predictions. The uncertainty estimates were then visualized alongside the predicted values, allowing for a comprehensive understanding of the model’s uncertainty and facilitating more informed decision-making based on the level of uncertainty.

**Figure 9:**
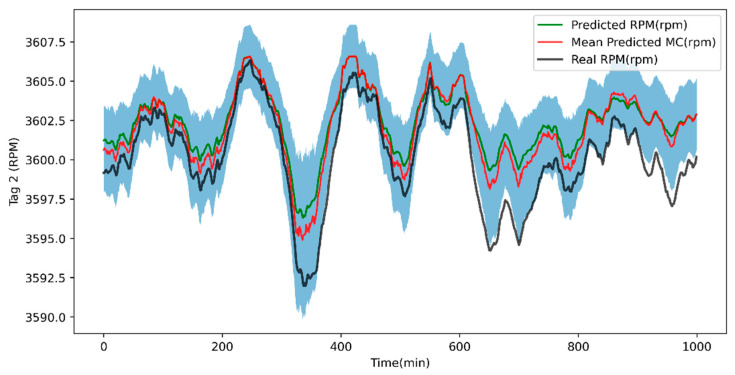
Rotational speed estimation and uncertainty evaluation.

Figure 9 shows the rotational speed estimation and its uncertainty evaluation by means of the Monte Carlo dropout simulation, considering a dropout (*p* = 0.25) and 1000 iterations.

Table 4 and Table 5 contain variations of the NARX recurring neural network hyperparameters that will be used to carry out the simulations associated with evaluating the uncertainties of the rotation speed of the gas turbine.

**Figure 10:**
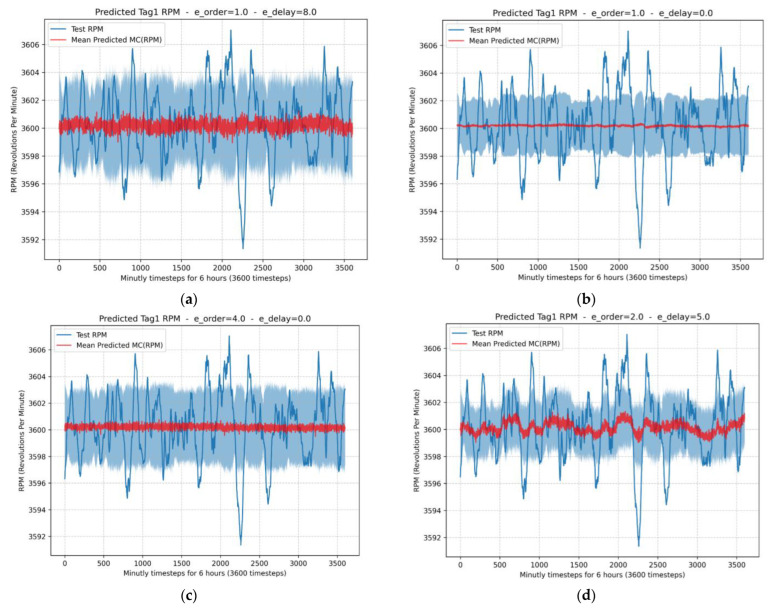
RPM estimation of the GT and uncertainty evaluation: (**a**) e_order = 1 and e_delay = 8; (**b**) e_order = 1 and e_delay = 0; (**c**) e_order = 4 and e_delay = 0; (**d**) e_order = 2 and e_delay = 5.

In Figure 10 shows the rotation speed estimation of the GT and the uncertainty evaluation for the different configurations presented in Table 6: (a) e_order = 1 and e_delay = 8; (b) e_order = 1 and e_delay = 0; (c) e_order = 4 and e_delay = 0; (d) e_order = 2 and e_delay = 5.

### 4.3. Analysis and Study of the Parameters Tuning of the Monte Carlo Dropout

This section will analyze the results obtained for the speed of rotation estimation of the developed model. Table 6 shows both the fixed values of the model, such as l, which was chosen arbitrarily. This parameter estimates the global uncertainty of the sensors used to acquire information. On the other hand, L2 regularization was used in order to avoid overfitting. This procedure is based on adding another term to the cost function of the model during training. It is used to control the complexity of the model and reduce the sensitivity to small variations in the training data. It helps to find a balance between fitting the training data too tightly and generalizing well to new data. For this reason, the regularization value will be linked to the confidence value of the model.

Thus, the Monte Carlo dropout technique was applied to calculate the maximum uncertainties of the model based on the standard deviation of each training sequence. Table 6 shows the results for different variations of *p*, *n_train*, and *M*. It can be seen that the values of the dropout probability were reduced, as well as the number of iterations of the model, affecting the reduction of the average standard deviation.

**Figure 11:**
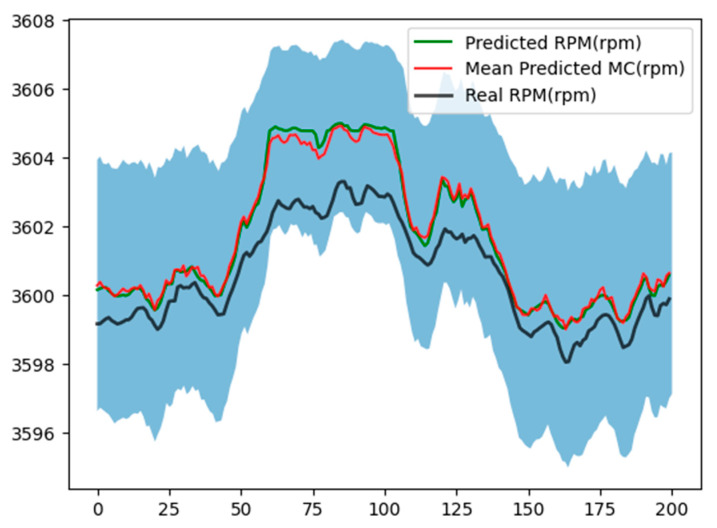
Monte Carlo dropout evaluation with M = 1000 and dropout probability of 0.25.

**Figure 12:**
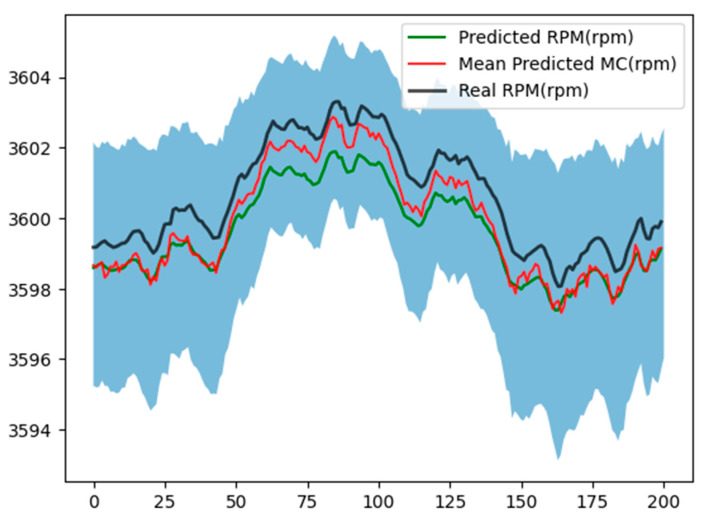
Monte Carlo dropout evaluation with M = 500 and dropout probability of 0.25.

Figure 11 and Figure 12 illustrate the uncertainties evaluation associated with the RPM estimation, considering 1000 and 500 interactions, respectively.

## 5. Conclusions

In this work, a methodology is developed for the estimation of the speed of the gas turbine using three topologies: NARX, NARX + LSTM, and LSTM. Based on the computational results, the best model obtained was the recurrent neural network (NARX + RNN) with a MAPE of 0.02%, which was the lowest value obtained compared to the MAPEs of the other proposed topologies.

The analysis of the evaluation of the uncertainties of the model based on the recurrent neural network NARX was carried out using the Monte Carlo dropout simulation method, in which it was verified that the uncertainty associated with the estimation of the rotation speed of the gas turbine depends on the parameters of the dropout probability and the number of iterations, configured in the Monte Carlo simulation. A minimum standard deviation of 2.2 RPM was obtained from the results obtained for a dropout probability of 0.05 and 500 iterations.

In this way, the proposed method can estimate the rotation speed of the gas turbine and simultaneously determine the associated uncertainty. This innovative method can be usefully used in models in which it is of interest to effectively control the estimation result of black-box models, such as models based on artificial neural networks.

In future work, the optimization of the set of hyperparameters associated with the uncertainty analysis is proposed (for example, the dropout probability and the number of iterations) using the Monte Carlo simulation method in order to optimize the confidence interval during the estimation of the speed of rotation of the gas turbine.

## Figures and Tables

**Table 1:** Variables of the GT.

Input Variables	Output Variables
*u*_1_(*t*): Gas fuel flow (Kg/s)	
*u*_2_(*t*): Inlet air temperature (°C)	*y*_1_(*t*): rotational speed (RPM)
*u*_3_(*t*): Barometric pressure (INH20)	

**Table 2:** Hyperparameters used in the NARX, NARX + LSTM, and LSTM models.

Model	NARX	NARX + LSTM	LSTM
Dense 1	32	32	128
Dropout	0.5	0.01	0.01
Dense 2	10	10	50
Batch Size	32	32	32
Activation function	Tanh	Tanh	Tanh
Lookback	5	5	15

**Table 3:** Evaluation metrics in the rotation speed estimation.

Model	NARX	NARX + LSTM	LSTM
MSE	1.80	5.22	7.62
MAE	0.98	1.78	2.15
MAPE	0.02%	0.045%	0.055%
Training time (s)	41.88	85.09	121.32

**Table 4:** NARX network parameter variations.

N° NARX	Dense 1	Dropout	Dense 2	Activation Function	Lookback
1	32	0.5	10	Tanh	5
2	32	0.25	10	Tanh	5
3	32	0.35	10	Tanh	15
4	128	0.15	32	Tanh	20
5	64	0.5	16	Tanh	5

**Table 5:** Variation of the training hyperparameters of the NARX network for its evaluation with Monte Carlo dropout.

N° NARX	Fixed Parameters			Flexible Parameters			
	l	λ	*n_Train*	*p*	*M*	e_Order	e_Delay
1	0.1	10^−6^	25	0.50	10	1	8
2	0.1	10^−6^	25	0.25	50	1	0
3	0.1	10^−6^	25	0.35	100	4	0
4	0.1	10^−6^	25	0.05	100	2	5
5	0.1	10^−6^	25	0.45	250	1	0

**Table 6:** Evaluation metrics in RPM estimation considering variations of the parameters of the Monte Carlo dropout simulation.

N° NARX	Fixed Parameters		Flexible Parameters			Results
	l	λ	*p*	*n_train*	M	Standard Deviation (MW)
1	0.1	10^−6^	0.25	250	500	2.64
2	0.1	10^−6^	0.25	250	1000	2.64
3	0.1	10^−6^	0.05	500	1000	2.21
4	0.1	10^−6^	0.05	500	500	2.19

## Data Availability

The private data presented in this study are available on request from the first author.
